# OVERWEIGHT IN ADOLESCENTS: FOOD INSECURITY AND MULTIFACTORIALITY IN SEMIARID REGIONS OF PERNAMBUCO

**DOI:** 10.1590/1984-0462/2020/38/2018177

**Published:** 2019-11-25

**Authors:** Natália Fernandes dos Santos, Pedro Israel Cabral de Lira, Fernanda Cristina de Lima Pinto Tavares, Vanessa de Sá Leal, Juliana Souza Oliveira, Jussara Tavares Pessoa, Poliana Coelho Cabral, Emília Chagas Costa

**Affiliations:** a Universidade Federal de Pernambuco, Recife, PE, Brazil.; b Universidade Federal de Pernambuco, Vitória de Santo Antão, PE, Brazil.

**Keywords:** Adolescent, Overweight, Obesity, Food supply, Socioeconomic factors, Cross-sectional studies, Adolescente, Sobrepeso, Obesidade, Segurança alimentar, Fatores socioeconômicos, Estudos transversais

## Abstract

**Objective:**

To investigate context of overweight adolescents from the semiarid and rural areas of Pernambuco, considering the multifactorial nature of the determinants of being overweight, and the food and nutritional insecurity conditions of the region.

**Methods:**

A population based cross-sectional study was conducted from September to October 2015. The nutritional status of adolescents was assessed by body mass index (BMI) and classified by the BMI/Age indicator, according to sex. To analyze the factors associated with being overweight, the variables were grouped into: socioeconomic, demographic, environmental, lifestyle, psychological, biological and food and nutritional security. Poisson regression was used to verify the association between being overweight and independent variables.

**Results:**

The prevalence of excessive weight found was 20.1%, namely: 13.4% overweight and 6.7% obese. After adjusting for the confounding variables, the variables: occupancy situation (rented house), alcohol consumption, food security and light food insecurity, body perception (overweight and obese) and age range (10 to 14 years), were associated with being overweight. High food and nutritional insecurity was identified in 80.4% of the population. The moderate and severe forms were more frequent, and precarious social conditions were still prevalent in the region.

**Conclusions:**

The prevalence of being overweight was high, exceeding the expected for a population with better living conditions. The determinants of being overweight were: alcohol consumption, occupancy situation, self-perceived weight, age and food security/mild food insecurity.

## RESUMO

**Objetivo:**

Investigar o excesso de peso em adolescentes do Sertão e Agreste de Pernambuco, considerando a multifatoriedade dos determinantes do excesso de peso e as condições de insegurança alimentar e nutricional da região.

**Métodos:**

Estudo transversal, de base populacional, realizado no período de setembro a outubro de 2015. O estado nutricional dos adolescentes foi avaliado pelo índice de massa corpórea (IMC) e classificado pelo indicador IMC/idade, segundo o sexo. Para analisar os fatores associados ao excesso de peso, as variáveis foram agrupadas em: socioeconômicas, demográficas, ambientais, de estilo de vida, psicológicas, biológicas e segurança alimentar e nutricional. A regressão de Poisson foi utilizada para verificar a associação entre o excesso de peso e as variáveis independentes.

**Resultados:**

A prevalência de excesso de peso encontrada foi de 20,1%, sendo 13,4% de sobrepeso e 6,7% de obesidade. Após o ajuste para as variáveis de confusão, as variáveis regime de ocupação (casa cedida, alugada), consumo de álcool, segurança alimentar, insegurança alimentar leve, percepção corporal (sobrepeso e obesidade) e faixa etária (10 a 14 anos) mostraram-se associadas ao excesso de peso. Destaca-se a elevada insegurança alimentar e nutricional, em 80,4% da população, sendo as formas moderadas e graves as mais frequentes, como também as precárias condições sociais ainda prevalentes na região.

**Conclusões:**

A prevalência de excesso de peso foi elevada, superando o esperado para uma população com melhores condições de vida. Os determinantes do excesso de peso foram: consumo de álcool, regime de ocupação, autopercepção do peso, faixa etária e segurança alimentar/insegurança alimentar leve.

## INTRODUCTION

Adolescence is a critical period for the development of obesity due to the physiological changes that occur during this phase, such as changes in body composition, in addition to hormonal, cognitive and emotional changes^1^ ^,^ ^2^. The consequences of obesity for adolescent health include dyslipidemia, hypertension, and glucose intolerance, as well as mental health effects such as depression and low self-esteem, and an increased chance of obesity in adulthood.^3^ ^,^ ^4^ Even in regions with high food and nutritional insecurity (INSAN), being overweight (OW) progressively reaches values similar to those in developed regions, due to the change from extreme qualitative and quantitative deprivation to an industrialized dietary pattern and sedentary lifestyle.^5^

The Northeastern Semiarid region is the poorest macroregion in Brazil, and includes the Sertão and Agreste municipalities of the state. It has a semiarid climate, and rainfall in this subregion is irregular and scarce, with constant periods of drought. The characteristics of this region result in serious economic and social consequences, with damage to agricultural production and acute food crises. In this regard, there are few studies evaluating the nutritional status of adolescents in the semiarid region. The last representative study in the region was the Nutritional Summons in the Semiarid Region (*Chamada Nutricional no Semiárido*)^5^ in 2005, which considered only children under five years old. No other study has assessed the living conditions and nutritional status of adolescents in the region. Searching the literature from 2007 to 2017, only seven studies evaluated the nutritional status of adolescents in the Sertão^6^ ^,^ ^7^ ^,^ ^8^ ^,^ ^9^ ^,^ ^10^ ^,^ ^11^ ^,^ ^12^, and only one in the Agreste region,^13^ which showed varied results. This highlights the evident gap in the nutritional surveillance of this population, and shows that depending on regional characteristics, the factors associated with nutritional changes may be different.

Considering the marked and progressive increase of being OW, the social consequences with regard to the health of affected individuals, as well as the many factors that lead to being OW, it is essential to understand this condition’s various aspects. Thus, the objective of this study was to investigate the prevalence of OW adolescents from the Sertão and Agreste (Semi-arid region) of Pernambuco, considering the multifactorial nature of the determinants of being OW, namely the social, demographic, biological, psychological and food and nutritional insecurity determinants of the region.

## METHOD

This was a population-based cross-sectional study that used data from two surveys using the same sample: “Assessment of food and nutrition security in urban and rural conglomerates affected by drought in the Pernambuco Sertão” and “Health, food, nutrition, services and socioeconomic conditions in the maternal and child population of the state of Pernambuco”. Both surveys were conducted from September to October 2015. The eligible population for the study consisted of all individuals between 10 and 19 years old, residing in the selected households. Adolescents who were pregnant or who had any functional limitation that compromised the anthropometric evaluation were excluded.

The sample size calculation considered the prevalence of being OW at 15%,^14^ with a maximum error of ± 5.5% and a confidence level of 95%, estimating a sample number of 162 individuals (plus 10% to cover eventual losses), totaling a sample size of 178 individuals. In order to estimate the associated factors, a confidence level of 95%, a study power of 80% and a ratio of exposed to unexposed of 1 to 1, based on an estimated risk of 2.7 were considered. The total was 184 individuals, 92 who were exposed and 92 who were unexposed.

**Figure 1:**
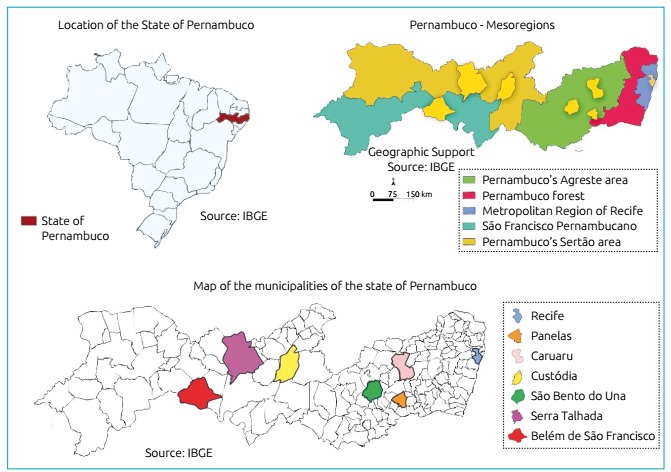
Map of where Pernambuco’s semi-arid municipalities are located.

The sample selection process (probabilistic and stratified) was carried out in four stages:

- Randomized selection of development macroregions, in which Pernambuco’s Agreste and Sertão territories were divided (Figure 1).
- Randomized selection of the municipalities to be included in the study.
- Randomized selection of census tracts (territorial units demarcated by the Brazilian Institute of Geography and Statistics - *Instituto Brasileiro de Geografia e Estatística* - IBGE).
- Randomized selection of households within each census tract to select households within a sample quota of 35 ± 5 households.

For the randomized selection of the municipalities and census tracts, we used the list of random numbers from the EPITABLE subprogram, Epi-Info™, version 6.04.

Data collection was performed by a team of technicians previously trained to apply the questionnaire and to measure anthropometric measurements, which followed the technical procedures recommended by the World Health Organization (WHO).^15^ Weight was obtained using a digital scale (TANITA Corporation, São Paulo, Brazil) with a capacity of 150 kg and an accuracy of 100 g. Height was measured using a portable stadiometer (Alturaexata Ltda., Belo Horizonte, Brazil) with an accuracy of 1 mm over the entire length. To ensure the accuracy of the measurements, they were taken twice for each individual, observing that the difference between the evaluations should not exceed 0.5 cm. To assess the nutritional status of the adolescents, the anthropometric index - body mass index ­(BMI)/­age was used. BMI was obtained by dividing body mass in kilograms by height in meters squared (kg/m^2^). The ­BMI/­age results were classified using the Anthro plus-2007 software and considered the WHO recommendations:^16^ thinness: <-2 Z score (EZ); eutrophic: ≥-2 EZ and ≤ +1 EZ; overweight: > +1 EZ and ≤ +2 EZ; obese: > +2 EZ.

The socioeconomic variables were collected in a specific survey questionnaire and included: socioeconomic class according to criteria of the Brazilian Association of Research Companies (*Associação Brasileira de Empresas de Pesquisa* - ABEP),^17^ education level of the head of household (years of schooling), education levels of the adolescent, occupancy situation, per capita income in minimum wage, participation in the Bolsa Família Program, number of rooms in the house, and number of people in the family.

The INSAN evaluation was performed by applying the Brazilian Food Insecurity Scale (*Escala Brasileira de Insegurança Alimentar* - EBIA).^18^ The questionnaire consists of 14 closed questions for households with residents under 18 years of age or 8 questions for households with residents over 18 years of age in addition to questions regarding the perception of respondents about the experience of not having enough food in the 3 months prior to the interview. Each affirmative answer on the questionnaire corresponded to one point and the sum of the points corresponded to the scale score. The sum of the scores was classified into four levels: 0 (zero)=food security; 1 to 5 points or 1 to 3 points=mild insecurity; 6 to 10 or 4 to 6 points = moderate insecurity; and 11 to 14 points or 7 to 8 points = severe insecurity for households with residents under 18 and over 18 years old, respectively.

Sexual maturity was determined based on the developmental stages proposed by Tanner,^19^ which included five stages for genital development (boys) and breasts (girls), evaluated for shape and size, as well as the assessment of pubic hair in both sexes. The adolescents were instructed to complete the questionnaire and were asked to perform a self-assessment. Based on the combination of the staging components, the adolescents were classified into three stages of maturation: stage 1 (E1) corresponding to the infantile phase (prepubescent); stage 2 (E2, E3, E4) corresponding to the pubescent phase; and stage 5 (E5), corresponding to the post-pubescent (adult) phase.^19^

The behavioral variables evaluated were: physical activity level, screen time, and alcohol consumption. Adolescents were considered to be active when they accumulated 300 minutes or more of weekly physical activity; insufficiently active when they accumulated from 1 to 299 minutes of physical activity per week; and inactive when they reported not practicing physical activity in the reference period.^20^ Excessive screen time was defined as the daily time spent watching television, playing a video game or using a computer, exceeding two hours per day.^21^ Alcohol consumption was considered when the adolescent answered that he or she had already tried it, and at the time of the interview, was still drinking it.

Self-perception of body weight was assessed using a questionnaire and included four response options: thin, normal, slightly overweight, and very overweight. Inadequate self-perception was considered when the response was in disagreement with the nutritional status diagnosis previously made by the BMI.^22^ Satisfaction with body weight was assessed by the question: “Are you satisfied with your body weight?” and the answer options were yes and no.

Data entry and verification was performed twice in the Validate module of the Epi-Info program, version 6.04 (*Centers for Disease Control and Prevention* - CDC, United States). For data processing and analysis, Epi-Info version 6.04, Statistical Package for Social Sciences (SPSS) version 13.0 (SPSS Inc., Chicago, IL, United States) and Stata version 7.0 (Stata Corp., College Station, United States) were used. For interpretation purposes, the type I error limit was 5% (p≤0.05).

To associate being OW with exposure variables, Pearson’s chi-square test and/or a linear trend were used, when applicable. For the analysis of the determinants of being OW, Poisson regression was performed, with a robust standard error option, in order to evaluate the adjusted effects of the component variables of the hierarchical model. Independent variables with p <0.20 in the bivariate analysis entered the multivariate analysis. In the hierarchical model, the socioeconomic and environmental variables constituted the most distal level, level 1; food and nutrition security, level 2; behavioral and psychological variables, level 3; and the biological variables, level 4, the most proximal level. The hierarchical analysis started with the level 1 variables and subsequently the variables of the other levels were introduced. Results were expressed as prevalence ratios (PR) adjusted with respective 95% confidence intervals (95%CI), with p<0.05 being considered statistically significant.

The study was approved by the Research Ethics Committee of the Health Sciences Center of the Universidade Federal de Pernambuco (CAAE: 38878814.9.0000.5208) and the Professor Fernando Figueira Institute of Integral Medicine (CAAE: 44508215.7.0000.5201), according to the ordinance no. 466/2012, of the National Health Council. The guardians signed a free and informed consent form and the adolescents signed a free and informed consent form.

## RESULTS

The sample consisted of 179 individuals of both sexes, with a median age of 14.7 years old, and an interquartile (IQ) range of 12.1-17.7. Of the total sample, the majority (67%) were female and over 90% were in the stage of pubertal sexual maturation. Almost half of the population lived in rural areas (48.6%) and had precarious socioeconomic situations (classes D and E; and per capita income up to 0.25 minimum wages). Socioeconomic vulnerability was also high (80.4%), with a majority of moderate and severe forms (48%). It is worth noting that 39.1% of adolescents showed inadequate education for age, represented by illiteracy or delays in the elementary and high school stages (Table 1).

**Table 1:** Overweight adolescents aged 10 to 19 years old according to socioeconomic and demographic variables. Sertão and Agreste, Pernambuco, 2015.

	Total		Overweight				p-value
			No		Yes		
	n=179	%	n=143	%	n=36	%	
Skin color							
White	53	29.6	43	81.1	10	18.9	0.34
Dark-skinned black	15	8.4	14	93.3	1	6.7	
Light-skinned black/others	111	62.0	86	77.5	25	22.5	
Food and nutritional security							
Light FNS/FNINS	93	52.0	70	75.3	23	24.7	0.10
Moderate/severe FNINS	86	48.0	73	89.4	13	15.1	
Economic class^#^							
B1, B2, C1	18	110.1	14	77.8	4	22.2	0.07
C2	72	40.2	52	72.2	20	27.8	
D, E	89	49.7	77	86.5	12	13.5	
Appropriate schooling for age							
Yes	108	60.3	88	81.5	20	18.5	0.48
No	70	39.1	54	77.1	16	22.9	
Education of the head of household							
Illiterate/ Incomplete ES	101	56.4	82	81.2	19	18.8	0.33
ES completed, MS incomplete, MS completed, HS incomplete	65	36.3	52	80	13	20.0	
HS completed, higher education incomplete and completed	13	7.3	9	69.2	4	30.8	
Job situation							
Works	47	26.3	38	80.9	9	19.1	0.84
Does not work	132	73.7	105	79.5	27	20.5	
Income per capita (in quartiles)							
Up to 0.25 MW	101	57.4	82	81.2	19	18.8	0.38
0.26 to 0.5 MW	52	29.5	43	82.7	9	17.3	
>0.5 MW	23	13.1	16	69.6	7	30.4	
Bolsa Família Program							
Yes	130	72.6	102	78.5	28	21.5	0.43
No	49	27.4	41	83.7	8	16.3	
Number of rooms							
1 to 4	130	14.5	102	78.5	28	21.5	0.43
5 to 10	49	85.5	41	83.7	8	16.3	
Number of people/family							
1 to 3	32	17.9	27	84.4	5	15.6	0.19
4 to 5	71	39.7	52	73.2	19	26.8	
≥6	76	42.5	64	84.2	12	15.8	

FNS: food and nutritional security (*segurança alimentar e nutricional*); FNINS: food and nutritional insecurity (*insegurança alimentar e nutricional*); ^#^economic class: criteria from the Associação Brasileira de Empresas de Pesquisa (2014); ES: elementary school; MS: middle school; HS: high school; MW: minimum wage. Totals differ due to losses in variables.

**Graph 1:**
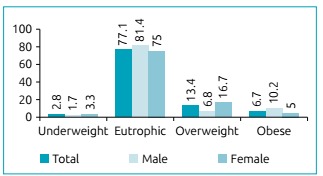
Nutritional status according to body mass index/age in adolescents aged 10 to 19 years old. Sertão and Agreste, Pernambuco, 2015.

The prevalence of being OW was high (20.1%), with 13.4% being overweight and 6.7% being obese (Graph 1). A height deficit was observed in 7.8% of the sample (data not shown).

In the bivariate analysis, the variables that were significantly associated with being OW were: place of residence, source of water supply, water treatment, physical activity level, body perception, body satisfaction and age range (Tables 1, 2 and 3). After adjusting for confounding variables, in the multivariate analysis, the factors that were associated with being OW were: occupancy (rented house), alcohol consumption, self-assessment of being obese, age between 10 and 14 years and light nutritional and food safety(SAN)/INSAN (Table 4).

**Table 2:** Overweight adolescents aged 10 to 19 years old according to environmental variables. Sertão and Agreste, Pernambuco, 2015.

	Total		Overweight				p-value
			No		Yes		
	n=179	%	n=143	%	n=36	%	
Household location							
Urban	92	51.4	66	71.7	26	28.3	0.005
Rural	87	48.6	77	88.5	10	11.5	
Occupancy situation							
Own	128	71.5	106	82.8	22	17.2	0.12
Rent	51	28.5	37	72.5	14	27.5	
Trash situation							
Collected	122	68.2	93	76.2	29	23.8	0.07
Burned, buried, other	57	31.8	50	87.7	7	12.3	
Source of water supply							
Public system	108	60.3	79	73.1	29	26.9	0.006
Well, cistern, other	71	39.7	64	90.1	7	9.9	
Water treatment							
Boiled, filtered, mineral	88	49.2	64	72.7	24	27.3	0.01
No treatment/other	91	50.8	79	86.8	12	13.2	
Sewage situation							
Public system, sump with a lid	139	79.0	109	78.4	30	21.6	0.47
Sump without a lid, water stream	37	21.0	31	83.8	6	16.2	
Floor type							
Ceramic	70	39.1	51	72.9	19	27.1	0.06
Cement, dirt, other	109	60.9	92	84.4	17	15.6	
Wall type							
Brick	167	93.3	134	80.2	33	19.8	0.66
Adobe and other materials	12	6.7	9	75.0	3	25.0	
Roof type							
Slab	14	7.8	11	78.6	3	21.4	0.89
Clay tile, asbestos	165	92.2	132	80.0	33	20.0	

Totals differ due to losses in variables.

**Table 3:** Overweight adolescents aged 10 to 19 years old according to biological, behavioral and psychological variables. Sertão and Agreste, Pernambuco, 2015.

	Total		Overweight				p-value
			No		Yes		
	n=179	%	n=143	%	n=36	%	
Sex							
Male	59	33.0	49	83.1	10	16.9	0.45
Female	120	67.0	94	78.3	26	21.7	
Age range (years)							
10 to 14	94	52.5	70	74.5	24	25.5	0.05
15 to 19	85	47.5	73	85.9	12	14.1	
Maturity level							
Prepubescent	15	8.6	14	93.3	1	6.7	0.18
Pubescent/Postpubescent	159	91.4	126	79.2	33	20.8	
Alcohol consumption							
Yes	27	15.1	19	70.4	8	29.6	0.18
No	152	84.9	124	81.6	28	18.4	
Physical Activity							
Yes	89	49.7	73	82.0	16	18.0	0.47
No	90	50.3	70	77.8	20	22.2	
Level of physical activity							
Active	18	10.1	10	55.6	8	44.4	0.006
Insufficiently active	71	39.6	63	88.7	8	11.3	
Inactive	90	50.3	70	77.8	20	22.2	
Screen time (during the week)							
<2 hours/day	64	35.8	48	75.0	16	25.0	0.22
≥2 hours/day	115	64.2	95	82.6	20	17.4	
Screen time (during the weekend)							
<2 hours/day	55	30.7	41	74.5	14	25.5	0.23
≥2 hours/day	124	69.3	102	82.3	22	17.7	
Weight perception							
Thin/normal	145	81.5	132	91.0	13	9.0	<0.001
Overweight/obese	33	18.5	11	33.3	22	66.7	
Body satisfaction							
Satisfied	111	62.0	99	89.2	12	10.8	<0.001
Unsatisfied	68	38.0	44	64.7	24	35.3	

Totals differ due to losses in variables.

**Table 4:** Crude and adjusted prevalence ratios for overweight adolescents from 10 to 19 years old. Agreste and Sertão, Pernambuco, 2015.

Variables	Overweight			p-value
	Crude PR (95%CI)	p-value	Adjusted PR (95%CI)	
Level 1				
Class				
B1, B2, C1,	1.07 (0.9-1.27)	0.07	1.02 (0.85-1.23)	0.46
C2	1.12(1.01-1.24)		1.09 (0.97-1.22)	
D, E	1.0		1.0	
Number of people/family				
4 to 5	1.09 (0.95-1.25)	0.20	1.08 (0.94-1.24)	0.68
≥6 people	1.00 (0.87-1.14)		1.00(0.87-1.14)	
1 to 3	1.0		1.0	
Household location				
Urban	1.15 (1.04-1.26)	0.003	1.01 (0.85-1.20)	0.69
Rural	1.0		1.0	
Occupancy situation				
Own	1.08 (0.97-1.21)	0.14	1.13 (1.04-1.24)	0.004
Rent	1.0		1.0	
Trash situation				
Collected	1.1 (0.99-1.2)	0.05	0.98 (0.87-1.11)	0.85
Burned, buried, other	1.0		1.0	
Source of water supply				
General system	1.15 (1.05-1.26)	0.002	1.06 (0.89-1.26)	0.49
Well, cistern, other	1.0		1.0	
Water treatment				
Boiled, filtered, mineral	1.12 (1.02-1.23)	0.01	1.06 (0.98-1.15)	0.13
No treatment/other	1.0		1.0	
Floor type				
Ceramic	1.09 (0.99-1.21)	0.06	1.04 (0.92-1.17)	0.531
Cement, dirt, other	1.0		1.0	
Level 2				
Nutritional security				
Light FNS/FNINS	1.08 (0.98-1.19)	0.10	1.08 (1.0-1.17)	0.03
Moderate/severe FNINS	1.0		1.0	
Level 3				
Alcohol consumption				
Yes	1.94 (0.94-1.26)	0.21	1.11 (1.01-1.20)	0.04
No	1.0		1.0	
Physical activity level				
Insufficiently active	0.77 (0.64-0.91)	0.006	0.90 (0.78-1.04)	0.83
Inactive	0.84 (0.71-1.00)		0.97 (0.84-1.11)	
Active	1.0		1.0	
Weight perception				
Overweight/obese	1.52 (1.37-1.70)	0.0001	1.45 (1.29-1.61)	<0.001
Thin/normal	1.0		1.0	
Body satisfaction				
Not satisfied	1.2 (1.10-1.34)	0.0001	0.97 (0.89-1.05)	0.87
Satisfied	1.0		1.0	
Level 4				
Age range				
10 to 14 years	1.1 (0.99-1.21)	0.051	1.09 (1.0-1.18)	0.03
15 to 19 years	1.0		1.0	
Maturity level				
Prepubescent	1.13 (0.99-1.28)	0.061	1.07 (0.92-1.23)	0.34
Pubescent/Postpubescent	1.0		1.0	

PR: prevalence ratio; 95%CI: 95% confidence interval; level 2: adjusted by level 1 variables; FNS: food and nutritional security; FNINS: food and nutritional insecurity; level 3: adjusted by the variables of modules 1 and 2; level 4: adjusted by the variables of modules 1, 2 and 3.

## DISCUSSION

In this study, we observed a high prevalence of being OW, which is lower than the national average, but exceeds the borderline value established by the WHO. Other studies conducted in the semiarid region of various parts of Brazil found the prevalence of being OW ranging from 10.5 to 41.1%.^5^ ^,^ ^6^ ^,^ ^8^ ^,^ ^9^ ^,^ ^13^ Our results were similar to those of the study conducted in the Semiarid region of Pernambuco in 2011 (25%)^13^ and lower than those observed in the Semiarid regions of the states of Rio Grande do Norte, Alagoas and Piauí (28.5 to 41.1%).^7^ ^,^ ^9^ ^,^ ^11^ This finding deserves attention, since it shows that even in populations with adverse social conditions, being OW is a progressively growing public health problem.

There is a tendency for the condition of being overweight to be concentrated in the least privileged social classes. Families of adolescents with lower socioeconomic status have a higher intake of processed foods, live in neighborhoods with little physical structure for physical activity, and care less about their physical appearance than young people from higher-class families.^23^ However, the association between social status and being OW depends on a number of factors such as gender, age and country of residence^13^ ^,^ ^14^. In this study, social status was not directly associated with being OW; variables such as family income and social class were not significantly associated. There was not enough contrast between the categories of these indicators to demonstrate such association, probably due to the widespread poverty of the population. On the other hand, the occupancy indicator of a rented house, which also reflects a worse social condition, was associated with being OW, perhaps indicating that this variable was more sensitive to identify the association of social condition of being OW. In fact, 49% of households with rented homes had moderate/severe INSAN, indicating that the quality of the food consumed was compromised and possibly affected adolescents’ weight.^23^

The WHO^20^ and the Brazilian Society of Pediatrics (*Sociedade Brasileira de Pediatria* - SBP) ^24^ recommend that school-age children practice at least 60 minutes of moderate to vigorous physical activity every day of the week, and have screen time that is less than two hours a day. In this study, it was found that 89.9% of adolescents are inactive or insufficiently active. When comparing studies with Brazilian adolescents that used the same cutoff as the present study to evaluate physical inactivity, the results obtained here were higher to those of the National School Health Survey (*Pesquisa Nacional de Saúde do Escolar* - PeNSE)^21^ (60.8% insufficiently active and 4.8% inactive) and that of the Cardiovascular Risks in Adolescents Study (*Estudo de Riscos Cardiovasculares em Adolescentes* - ERICA)^25^ (54.3% inactivity). The high frequency of physical inactivity in this study could, in part, be explained by the unfavorable environmental characteristics of physical activity in the Semiarid region, such as the warm climate, the level of urbanization, and the poor infrastructure. In this region there are few community recreational spaces such as parks, bike paths and jogging/walking paths that, if present, could stimulate a more active life.

The prevalence of excessive screen time in this study was high (64.2 to 69.3%), higher than that observed in a survey of students from the Northeast,^26^ who evaluated sedentary leisure (66.1 to 57.8%). A recent systematic review reveals that two or more hours of television per day are associated with various health hazards, such as high body weight, decreased physical fitness, low self-esteem, and poor student performance.^27^ The data found confirm the globalization trend of the Semiarid region. The population follows the behavior patterns of large urban centers, with a sedentary lifestyle and possible alterations in the eating patterns.

In our study, active individuals had a higher frequency of being OW. This contradictory result could be explained by the phenomenon of reverse causality characteristic of cross-sectional studies. Individuals may have started practicing physical activity after becoming obese, but by the time of the survey, there would not have been enough time for physical activity to show the effects of reducing obesity.

Sexual maturity in this study was not associated with being OW. Studies evaluating this association generally consider the delay or earliness of this process. In adolescence, the pubescent stage influences anthropometric and body composition parameters, but due to the variability of sexual maturity according to sex and age, and the limitations of the maturity classification method, such a relationship is not always established in studies.^28^ Regarding chronological age, there was a tendency to decrease the prevalence of being OW and physical activity with the progressive increase of age. In early adolescence, individuals are more susceptible to peer and media influence, which would provide for being OW. Adolescents in their late teens who have already developed their personal identities become less likely to be influenced, as they can reflect on the consequences of their habits and choices, and they are more concerned with their physical appearance.^29^

Alcohol consumption was associated with an 11% higher chance for being OW. Other studies confirm that, compared to young people with a normal weight, adolescents that are OW are more likely to be frequent drinkers or to start consuming alcohol early.^30^ While frequent use of alcohol, which is a considerable caloric source, can lead a young person of normal weight to become OW, an obese teenager can become a frequent drinker. Adolescents that are OW often have difficulty making friends and experience discrimination. Obese adolescents tend to choose similarly marginalized peers, combining peer-based influences for risky behavior such as smoking, alcoholism and restrictive diets. They resort to these behaviors in order to change their body shape and be accepted. ^30^ In fact, of overweight adolescents, only 10.8% were satisfied with their body, while those that were not overweight, body satisfaction was 89.2%.

A comparison between BMI categories and self-perception of body weight showed that among adolescents classified as being OW, 33.3% underestimated their nutritional status. It is likely that the low socioeconomic status and the low educational level of the parents influenced the results of the adolescents’ body perception. Less educated parents often underestimate the weight of their children because they tend to associate being overweight with better health, while young people with better socioeconomic status are more pressured to fit the ideal of slim beauty.^31^ Adolescents’ self-rating if themselves as being OW was associated with a 45% higher chance of actually being obese. Adolescents and parents’ recognition of obesity may be the first step in their treatment. On the other hand, when the adolescent or parents do not recognize obesity, the search for health professionals may be too late, increasing the chances that the adolescent does not adequately perform the prescribed treatment. ^31^

INSAN in this study totaled 80.4% of the population, with moderate and severe forms being the most frequent. This result is similar to the study performed by Oliveira,^6^ with adolescents from a city in the Northeastern Semiarid region, which found an INSAN prevalence of 91.5%. In economic terms, the Northeastern Semiarid region is the poorest macroregion in Brazil, having a majority of the poor population. Its characteristics of irregular climate and poor soils have severe economic and social consequences, with losses in agricultural production and acute food crises. Adolescents that are in SAN or mild INSAN had a higher chance of being OW. SAN/INSAN conditions may reflect better conditions for the purchasing of food, even if it is unhealthy. On the other hand, moderate and severe INSAN reflects hunger or the difficulty in accessing or purchasing food in sufficient quantities for one’s nutritional needs. A second rather surprising aspect is the coexistence of a high INSAN with a low prevalence of malnutrition and a high prevalence of being eutrophic. This nutritional paradox is considered a small moment in the epidemiological transition process that is occuring in Brazil.^6^

In 2005, the Semiarid Nutritional Summons^5^ assessed the nutritional status and social conditions of children under the age of five in the Semiarid region and settlements in the northeast and north of Minas Gerais. This survey revealed the adverse socioeconomic conditions of households in Pernambuco: the prevalence of classes D and E of 41.6 and 33.3%, respectively; illiteracy and low levels of education in 12.7 and 30.1% of heads of households, respectively; and a public water supply system in 76.7% of the households. In the present study, the majority of the families of the adolescents evaluated belonged to classes D and E (49.7%), the majority of the heads of the family had little or no education (56.4%) and the source of water was unsatisfactory, with only 60.3% of the water supply coming from the public system. Regarding family income, there was no significant difference in the bank statements, which may be due to the homogeneity of the sample. On the other hand, adolescents living in a rented house were associated with a higher risk of being OW. Occupancy is an indicator of social status, suggesting that worse living conditions are associated with being OW.

The present study presented several limitations. First, its cross-sectional nature, which does not make it possible to test causal relations; the lack of inclusion of food intake data and body composition measures; and the use of a questionnaire to assess physical activity that did not allow for the intensity, frequency and duration of physical activities to be determined. Data were collected in a specific region of the country and therefore generalization of these results to other regions should be done with caution.

Despite the limitations, the present study brings relevant contributions to the table by investigating the prevalence of being OW in an adolescent population in the Semiarid region, which are scare in the literature. Regarding the methodological aspects, the sample was probabilistic and data quality was guaranteed by conducting a pilot study, training interviewers, checking questionnaires and entering the data twice. Measurements were measured using appropriate techniques, scientific instruments and qualified evaluators. Additionally, few studies evaluate being OW with the broad spectrum of variables that were used in this study, which contemplates the multiplicity of risk factors that the condition of being OW presents.

The prevalence of being OW in adolescents in the Semiarid region was high, although the population presented high socioeconomic vulnerability and food insecurity. Factors associated with being OW were: alcohol consumption, occupancy, self-perceived weight, age and mild SAN/INSAN. The aim of this study is to warn of the need for public policies to combat poverty and the condition of being overweight in these poorer areas of the northeast region
